# Dynamic Experiment Design Regularization Approach to Adaptive Imaging with Array Radar/SAR Sensor Systems

**DOI:** 10.3390/s110504483

**Published:** 2011-04-27

**Authors:** Yuriy Shkvarko, José Tuxpan, Stewart Santos

**Affiliations:** Department of Telecommunications of Center of Research and Advanced Studies of I.P.N., Av. del Bosque 1145, Col. El Bajío, Zapopan, Jalisco, C.P. 45019, México; E-Mails: jtuxpan@gdl.cinvestav.mx (J.T.); ssantos@gdl.cinvestav.mx (S.S.)

**Keywords:** adaptive sensing, experiment design, radar imaging, sensor system, spatial spectrum pattern (SSP), synthetic aperture radar (SAR), regularization, variational analysis

## Abstract

We consider a problem of high-resolution array radar/SAR imaging formalized in terms of a nonlinear ill-posed inverse problem of nonparametric estimation of the power spatial spectrum pattern (SSP) of the random wavefield scattered from a remotely sensed scene observed through a kernel signal formation operator and contaminated with random Gaussian noise. First, the Sobolev-type solution space is constructed to specify the class of consistent kernel SSP estimators with the reproducing kernel structures adapted to the metrics in such the solution space. Next, the “model-free” variational analysis (VA)-based image enhancement approach and the “model-based” descriptive experiment design (DEED) regularization paradigm are unified into a new dynamic experiment design (DYED) regularization framework. Application of the proposed DYED framework to the adaptive array radar/SAR imaging problem leads to a class of two-level (DEED-VA) regularized SSP reconstruction techniques that aggregate the kernel adaptive anisotropic windowing with the projections onto convex sets to enforce the consistency and robustness of the overall iterative SSP estimators. We also show how the proposed DYED regularization method may be considered as a generalization of the MVDR, APES and other high-resolution nonparametric adaptive radar sensing techniques. A family of the DYED-related algorithms is constructed and their effectiveness is finally illustrated via numerical simulations.

## 1. Introduction

Space-time adaptive processing (STAP) for high-resolution radar imaging with sensor arrays and synthetic aperture radar (SAR) systems has been an active research area in the environmental remote sensing (RS) field for several decades, and many sophisticated techniques are now available (see among others [1–4] and the references therein). The problem of radar/SAR imaging can be formalized in terms of nonlinear inverse problems of nonparametric estimation of the power spatial spectrum pattern (SSP) of the random wavefield scattered from the remotely sensed scene observed through a kernel signal formation operator (SFO) with the kernel structure specified by the employed radar/SAR signal modulation and contaminated with random Gaussian observation noise [1,2,5]. Thus, formally, the RS imaging problem falls into a category of stochastic ill-posed nonlinear inverse problems. The simplest radar/SAR-oriented robust approach to such the problem implies application of a method known as “matched spatial filtering” (MSF) to process the recorded data signals [1–3]. Stated formally [2,3] the MSF method implies application of the adjoint SFO to the recorded data, squared detection of the filter outputs and their averaging over the actually recorded samples (snapshots) [1] of the independent data observations. One of the challenging aspects of the array radar/SAR imaging relates to development of high-resolution efficient consistent STAP techniques applicable to the scenarios with low number of recorded array snapshots (one snapshot data vector as a limiting case) or only one recorded realization of the trajectory data signal in a SAR system. In both cases, the data sample covariance matrix is rank deficient (rank-1 in the single look SAR case), and none of the conventional nonparametric beamformers [6–9], nor the maximum likelihood (ML) related high-resolution STAP techniques [1,4,7–11] are able to produce consistent SSP estimates. Moreover, speckle noise and possible array/SAR calibration errors constitute additional multiplicative sources of data degradations that inevitably aggravate the problem inconsistency resulting in the heavily distorted speckle-corrupted scene images. In addition, because the real-world RS scenes are implicitly associated with distributed inhomogeneous fields (*i.e*., not composed of a small number of point-type targets), none of the recently developed sparsity-based techniques such as independent component analysis [12], principal component analysis [13] or kernel independent component analysis [14] are able to cope with such type of ill-conditioned RS imaging problems. To alleviate the inconsistency (and to perform adaptive image despeckling [5,15]), another group of the variational analysis (VA) related methods that fall into the category of the so-called “blind” or “model-free” image enhancement approaches have recently been adapted to RS image enhancement, e.g., [16–20] but without their aggregation with the resolution enhancing “model-based” nonparametric regularized imaging techniques [21–24].

Another possible way to alleviate the ill-posedness of the nonlinear radar/SAR imaging problems is to incorporate a priori model considerations regarding the desired geometrical scene image properties into the STAP procedures via performing randomization of the SSP model and application of the Bayesian minimum risk (MR) or maximum *a posteriori* probability (MAP) nonparametric adaptive spatial spectral estimation strategies [3,21]. Unfortunately, such approaches lead to the nondeterministic polynomial-type (NP) hard computational procedures [21], and hence result in technically unrealizable SSP estimators. An alternative way that we propose and describe in this study is to incorporate (as the second dynamic regularization level) the anisotropic kernel window operator (WO) into the overall descriptively regularized ML-based iterative adaptive SSP estimator and perform projections onto convex sets (POCS) that ensure the consistency and at the same time enforce the convergence of the resulting doubly regularized adaptive iterative imaging procedures. First, we adapt the most prominent recently proposed nonparametric ML inspired amplitude and phase estimation (APES) method (ML-APES method) [24] to the imaging problem at hand following the descriptive experiment design (DEED) regularization paradigm [25,26]. Second, to transform the DEED-optimized adaptive nonlinear imaging technique into the iterative convergent procedure the POCS regularization is employed. To guarantee the consistency, the anisotropic kernel WO is incorporated into the composed POCS operator adjusted to the metrical properties of the desired images in the Sobolev-type solution (image) space. Thus, the “model-free” variational analysis (VA)-based image enhancement approach [16–20] and the “model-based” descriptive experiment design (DEED) regularization paradigm [25,26] are unified into a new dynamic experiment design (DYED) regularization framework. Application of the proposed DYED framework to the high-resolution array radar/SAR imaging problems leads to a class of two-level (DEED-VA) regularized SSP reconstruction techniques that aggregate the anisotropic kernel adaptive dynamic processing with projections onto convex sets to enforce the consistency and convergence of the overall iterative SSP estimators. We also show how the proposed DYED regularization approach may be considered as a generalization of the APES [24], and some other novel high-resolution “model-based” nonparametric radar imaging techniques [15,27], on one hand, and the VA-related anisotropic diffusion [16,18], selective anisotropic information fusion [20] and other nonparametric “model-free” robust adaptive beamforming based image enhancement approaches [28–33], on the other hand.

The reminder of the paper is organized as follows. In Section 2, we provide the formalism of the radar/SAR inverse imaging problem at hand with necessary experiment design considerations. In Section 3, we compare the ML-APES approach with the DEED-related family of the SSP estimators. The performance guarantees are conceptualized in Section 4. An extension of the VA-based dynamic POCS regularization unified with the DEED paradigm that results in a new proposed DYED framework is addressed in Section 5 followed by some illustrative simulations and discussion in Sections 6 and conclusions in Section 7, respectively.

## 2. Background

The general mathematical formalism of the problem at hand and the DEED regularization framework that we employ in this paper are similar in notation and structure to that described in [10,11,25,34] and some crucial elements are repeated for convenience to the reader.

### 2.1. Problem Formalism

In a general continuous-form (functional) formalism, a random temporal-spatial realization of the data field, *u*, is considered to be created by some continuous distribution of the far-distant radiation/scattering sources *e* as plane or spherical wavefronts, which sweep across the radar sensor array (moving antenna in the case of SAR). These fields satisfy an operator-form linear stochastic equation (the so-called *equation of observation* (EO) [10,21]):

(1) u(p)=(𝒮e(r))(p)+n(p);            e(r) ∈ 𝔼(R);           u(p),  n(p) ∈ 𝕌(P)

where **p** = (*t*, **ρ**) defines the time (*t*)—space (**ρ**) points in the temporal-spatial observation domain **p** ∈ *P* = *T*× P (*t* ∈ *T*, **ρ** ∈ P) (in the SAR case, **ρ** = **ρ**(*t*) specifies the carrier trajectory, *i.e.*, the array is composed of the moving antenna); *e*(**r**) represents the random scene reflectivity over the probing surface; **r** is a vector of the scan parameters, usually the polar, cylindrical or Cartesian coordinates of the probing surface *R*; *n* corresponds to the additive noise field, and the linear kernel SFO 𝒮: 𝔼(*R*) → 𝕌(*P*) defines a mapping of the source signal space 𝔼(*R*) onto the observation signal space 𝕌(*P*). The metrics structures in the corresponding Hilbert signal spaces 𝕌(*P*) and 𝔼(*R*) are imposed by scalar (inner) products:

(2) $${\left[u,u^{\prime }\right]}_{𝕌}=\int_{P}u(p)u^{\prime }*(p)dp,\;\;\;\;\;\;\;\;\;{\left[e,e^{\prime }\right]}_{𝔼}=\int_{R}e(r)e^{\prime }*(r)dr$$

respectively, where asterisk stand for complex conjugate. In the conventional integral form, EO (1) may be rewritten as:

(3) $$u(p)=\int_{R}S(p,r)e(r)dr+n(p),$$

where *S*(**p**, **r**) = *S*(*t*, **ρ**; **r**) represents the functional kernel of the SFO referred to as the *unit signal* [10,34] determined by the time-space modulation employed in a particular RS system, and the scene domain *R* specifies the bounded SFO support. For explicit definitions of the unit signals for array radar systems we refer readers to to [7], (Sec. 6, [21]) and for imaging SAR systems to [4,22], (Sec. 5, [11]) and (Sec. 6, [26]).

It is convenient in the RS applications to assume that due to the integral signal formation model (3), the central limit theorem conditions hold [3,23,34,35], hence the fields *e*, *n*, *u* in (1), (3) are considered to be the zero-mean complex-valued random Gaussian fields. Next, since in all RS applications the regions of high correlation of *e*(**r**) are always small in comparison with the resolution element on the probing scene [3,10,11,34], the signals *e*(**r**) scattered from different directions **r**, **r**′ ∈ *R* are assumed to be uncorrelated, *i.e.*, characterized by the correlation function:

(4) $$R_{e}(r,\;r^{\prime })=〈e(r)e*(r^{\prime })〉=b(r)\delta (r-r^{\prime });\;\;\;\;r,r^{\prime }\in R$$

where *δ*(·) defines the delta function and 〈·〉 is the averaging (expectation) operator. The average

(5) $$b(r)=〈e(r)e*(r)〉=〈|e(r){|}^{2}〉;\;\;\;r\in R$$

of the square modules of the random scattering field *e*(**r**) as a function over the analysis domain (scene frame) *R*∋**r** has a statistical meaning of the average power scattering function and is traditionally referred to (in the RS and radar imaging literature, e.g., [1,4,31,32,34], *etc.*) as the *SSP* of the scattered field. Representing the spatial distribution of the average power of the random scatterers, the SSP *b*(**r**) characterizes in an explicit statistical sense the brightness reflectivity of the scene being mapped (for this reason, *b* is adopted in the notations as an abbreviation from *brightness reflectivity*). The estimate *b̂* (**r**) of the SSP formed using some statistically grounded method is associated with the scene image to be formed via processing the recorded data observations.

### 2.2. Experiment Design Considerations

The formulation of the data discretization and sampling in this paper follows the experiment design formalism given in [10,23,30,34] that enables one to generalize the finite-dimensional approximations of Equations (1,3) independent of the particular system configuration and the method of data measurements and recordings employed. Following [10,34], consider the sensor array (synthesized array) specified by a set of distanced in space (*i.e.*, orthogonal) tapering functions $\left\{\kappa_{l}^{*}(\rho );l=1,\ldots ,L\right\}$ (in the SAR case, the $\left\{\kappa_{l}^{*}(\rho )\right\}$ are synthesized by the moving antenna over *L* spatial recordings [4,34]). Consider next, that the output signals in such spatially distributed measurement channel are then converted to *I* samples at the outputs of identical temporal sampling filters defined by their impulse response functions $\left\{v_{i}^{*}(t);i=1,\ldots ,I\right\}$ where complex conjugate is taken for notational convenience. Without loss of generality [2–4,10,21,34], the sets {*κ_l_*} and {*v_i_*} are assumed to be orthonormal (e.g., via proper filter design and sensor antenna calibration [4,10]). The composition {*h_m_*(***p***) = *κ_l_*(*ρ*)*v_i_*(*t*); *m* = (*l*, *i*) =1,..., *L* × *I* = *M*} ordered by multi-index *m* = (*l*, *i*) composes a set of the orthonormal spatial-temporal decomposition functions (base functions) that explicitly determine the vector of outcomes:

(6) $$u=\underset{m}{vec}\left\{u_{m}={\left[u,\;h_{m}\right]}_{𝕌}=\int_{P}u(p)h_{m}^{*}(p)dp;\;\;\;\;m=1,\ldots ,M\right\}$$

of such an *M*-dimensional (*M*-D in our notation) data recording channel, in which the employed base functions {*h_m_*(**p**)} span the relevant *M*-D data representation subspace 𝕌_(_*_M_*_)_ = 𝒫_𝕌(_*_M_*_)_ 𝕌 = Span_(_*_M_*_)_{*h_m_*(**p**)} specifying the corresponding projection operator 𝒫_𝕌(*M*)_ defined by Equation (6).

In analogy to Equation (6), one can define now the *K*-D vector-form approximation of the scene random scattering field:

(7) $$e=\underset{k}{vec}\left\{e_{k}={\left[e,\;g_{k}\right]}_{𝔼}=\int_{R}e(\text{r})g_{k}^{*}(r)dr;\;\;\;\;\;k=1,\ldots ,K\right\}$$

with the elements composed of the decomposition coefficients {*e_k_*} with respect to some chosen normalized set of expansion functions {*g_k_*(**r**)} that span such *K*-D source signal subspace 𝔼_(_*_K_*_)_ = 𝒫_𝔼(_*_K_*_)_ 𝔼 = Span_(_*_K_*_)_{*g_k_*(**r**)} specifying the corresponding projector 𝒫_𝔼(_*_K_*_)_. Note, that to satisfy the observability requirement [2,21], it is desirable (but not mandatory in the super-resolution case [22,24]) that these basis functions be properly adjusted in such a way that 𝒫_𝔼(_*_K_*_)_ defines a projector onto the *M*-D orthogonal complement to the null-space [2] of the degenerate (projection-dependent) SFO 𝒫_𝕌(_*_M_*_)_𝒮 for any given/chosen 𝒫_𝕌(_*_M_*_)_ so that the observations (6) contain information of the observable *M*-D signal projection specified by Equation (7), *i.e.*, *K* ≤ *M*. The reader is referred to [2,10,21,22,24,25,34] for the mathematical and signal processing details related to the construction of such feasible projectors 𝒫_𝕌(_*_M_*_)_ and 𝒫_𝔼(_*_K_*_)_. In this paper we adopt the technically inspired fine representation basis formed by a *K_x_*×*K_y_* regular pixel-formatted lattice with unitary pixel amplitudes and the spacing between lattice points normalized to one pixel width [2,25,34] where *K_x_* defines the dimension of the rectangular pixel grid over the horizontal (azimuth) coordinate *x* and *K_y_* defines its dimension over the orthogonal (range) coordinate *y* in the scene frame. Note that such rectangular pixel frame {pix*_k_*(**r**)} specified by the ordered multi-index *k* = (*k_x_*, *k_y_*); *k_x_*_=_ 1,.., *K_x_*; *k_y=_* 1,.., *K_y_*; *k* = 1,…, *K* = *K_x_* × *K_y_* is practically motivated in a majority of RS imaging applications [1,3–11,18,26,34,37], *etc*, because the ordinary pixels coincide with their squares $\left\{{\text{pix}}_{k}(r)={\text{pix}}_{k}^{2}(r)\right\}$ which makes the same pixel grid {*g_k_*(**r**) = pix*_k_*(**r**)} applicable for fine discrete-form representation of both the complex scattering function *e*(**r**) and the SSP *b*(**r**) approximating for large *K_x_*, *K_y_* the continuous scene framing [2].

With the specified decompositions (6), (7), the discrete (vector-form) approximation of the continuous-form EO (1), (3) is given by:

(8) u=Se+n,

where the **u**, **n** and **e** define the vectors composed of the decomposition coefficients *u_m_*, *n_m_* and *e_k_* of the finite-dimensional (truncated) approximations of the fields *u*, *n* and *e* defined by the scalar products (6), (7), and **S** represents the matrix-form approximation of the SFO 𝒮 with elements [21]:

(9) $$\left\{S_{mk}={\left[𝒮g_{k},\;h_{m}\right]}_{𝕌}=\iint_{R\times P}S(p,\;r)g_{k}(r)h_{m}^{*}(p)drdp;\;\;\;\;\;\;\;\;\;\;\;\;\;\;\;\;k=1,\ldots ,K;\;\;\;m=1,\ldots ,M\right\}.$$

Zero-mean Gaussian vectors **e**, **n**, and **u** in Equation (8) are characterized by the correlation matrices **R_e_**, **R_n_**, and:

(10) $$R_{u}=SR_{e}S^{+}+R_{n}=\text{SD}(b)S^{+}+R_{n}$$

respectively, with the diagonal-form **R_e_** = **D**(**b**) = diag(**b**), in which the *K* × 1 vector **b** of the principal diagonal is composed of elements $b_{k}=〈e_{k}e_{k}^{*}〉$ and the superscript ^+^ defines the Hermitian conjugate when stands with a matrix (or a vector). The vector **b** is referred to as a vector-form representation of the SSP, *i.e.*, the SSP vector.

The nonlinear inverse problem of radar/SAR imaging with the discrete-form measurement data (8) is formulated now as follows: to derive an estimator for the SSP vector **b** and use it to reconstruct the SSP distribution:

(11) $${\hat{b}}_{\left(k\right)}=\text{est}\{〈|e_{\left(k\right)}(r){|}^{2}〉\}=\sum_{k=1}^{K}{\hat{b}}_{k}g_{k}(r)$$

over the pixel-formatted observation frame *R* ∋ **r** (referred to as the scene image) by processing the recorded data **u** (in the operational scenario with the single processed data realization, e.g., SAR system) or *J* > 1 whatever available independent realizations {**u**_(_*_j_*_)_; *j* = 1,.., *J*} (e.g., in a multiple snapshot scenario [5,15]) collected with a particular imaging radar/SAR system. Recall that in this paper we intend to develop and follow a new DYED framework to derive such the estimator est{·} (11) that unifies the previously proposed DEED regularization with the VA dynamic image enhancement approach.

### 2.3. Conventional Kernel Spectral Estimator

We, first, recall the conventional continuous-form kernel estimator [2,21] that is an MSF-based extension of the periodogram smoothing spectral analysis technique [36,37]. With such the method, the SSP estimate *b̂*(**r**) is derived from only one random observation (realization) of the data field *u*(**p**) as the generalized periodogram (the so-called *sufficient statistics* [21]) formed as a squared modulus of the MSF output | (𝒮^+^*u*)(**r**)|^2^ smoothed by the kernel window operator (WO) 𝒲 (*i.e.*, pseudo averaged):

(12) $$\hat{b}(r)=𝒲|({𝒮}^{+}u)(r){|}^{2}=\int_{R}W(r-r^{\prime }){\left|\int_{P}S*(r^{\prime },\;p)u(p)dp\right|}^{2}dr^{\prime },$$

where *W*(**r**) is the functional kernel of the WO 𝒲, and S^*^ (**r**′, **p**) represents the functional kernel of the MSF operator 𝒮^+^, that is, the adjoint SFO. Here, superscript + stands for the adjoint operator in the relevant signal spaces. In the Hilbert signal spaces introduced above, the MSF operator 𝒮^+^ adjoint to the SFO 𝒮 is defined via corresponding inner products (2) as follows, [*e*,𝒮^+^*u*]_𝔼_ = [𝒮*e*,*u*]_𝕌_. For a detailed analysis of this method and the corresponding synthesis of different windows with special scaling and smoothing properties we refer to [36]. Note that in a classical case of an isotropic kernel WO, the Equation (12) relates to a category of low-resolution kernel-type SSP estimators.

## 3. Related Work

### 3.1. ML-Based Approach

In this Section, we extend the recently proposed high-resolution maximum likelihood-based amplitude phase estimator (ML-APES) [24] to the SSP estimation problem at hand via its modification adapted to the distributed RS scene (not composed of sparse multiple point-type targets as originated in [24]). In the considered low snapshot sample case (e.g., one recorded SAR trajectory data signal), the sample data covariance matrix $Y=(1/J)\sum_{j=1}^{J}u_{\left(j\right)}u_{\left(j\right)}^{+}$ is rank deficient (rank-1 in the single radar snapshot and single look SAR cases, *J* = 1). As it is shown in [24], minimization of the negative likelihood function $\text{lndet}\{R_{u}\}+\text{tr}\{R_{u}^{-1}Y\}$ with respect to the SSP vector **b** related to **R_u_** = **R_u_**(**b**) via Equation (10) is equivalent to minimizing the covariance fitting Stein’s loss, $-\text{lndet}\{R_{u}^{-1}Y\}+\text{tr}\{R_{u}^{-1}Y\}$. The solution to such minimization problem found in [24] results in the solution-dependent ML-APES estimator ([24], Equation (32)):

(13) $${\hat{b}}_{k}=\frac{s_{k}^{+}R_{u}^{-1}YR_{u}^{-1}s_{k}}{{\left(s_{k}^{+}R_{u}^{-1}s_{k}\right)}^{2}};\;\;\;k=1,\ldots ,K.$$

In the APES terminology (as well as in the minimum variance distortionless response (MVDR) [9,33] and other ML-related approaches [23,35], *etc.*), **s***_k_* represents the so-called *steering* vector in the *k*th look direction, which in our notational conventions is essentially the *k*th column vector of the SFO matrix **S**. By the authors’ design [24], the numerical implementation of the ML-APES algorithm (13) assumes application of an iterative fixed-point technique by building the model-based estimate **R̂_u_** = **R_u_** (**b̂**_[_*_i_*_]_) of the unknown covariance **R_u_** modeled by Equation (10) from the latest (*i*th) iterative SSP estimate [**b̂**_[_*_i_*_]_] with the zero step initialization **b̂**_[0]_ = **b̂***_MSF_* computed applying the conventional MSF estimator.

Let us adapt the algorithm (13) to the considered here single snapshot/single look case (*J* = 1) substituting **Y** by **uu**^+^, taking into account the properties of the convergent MVDR estimates of the SSP, which in a coordinate/pixel form are given by ${\hat{b}}_{k}\approx 1/s_{k}^{+}R_{u}^{-1}s_{k}$ (also referred to as properties of a conventional Capon beamformer [9,24]), and making the use of a fixed-point nature of the algorithm (13) according to which the ML-APES estimates in the vector form $\hat{b}=\underset{k}{vec}\{{\hat{b}}_{k};k=1,\ldots ,K\}$ are to be found as a numerical solution to the nonlinear matrix-vector equation:

(14) $${\hat{b}}_{\text{ML-APES}}={\left\{\hat{D}S^{+}R_{u}^{-1}(\hat{b})uu^{+}R_{u}^{-1}(\hat{b})S\hat{D}\right\}}_{\text{diag}}$$

with the solution-dependent:

(15) $$\hat{D}=D(\hat{b})=\text{diag}(\hat{b})\;\;\;\;\;\;\;\;\;\;\;\;\;\text{and}\;\;\;\;\;\;\;\;\;\;\;\;\;R_{u}^{-1}(\hat{b})={\left(S\hat{D}S^{+}+R_{n}\right)}^{-1}$$

where operator {·}_diag_ returns the vector of a principal diagonal of the embraced matrix. Specifying the ML-APES matrix-form solution operator (SO):

(16) $$F_{\text{APES}}=F^{\left(1\right)}=\hat{D}S^{+}R_{u}^{-1}(\hat{b}),$$

we next represent the estimate (14) in a more compact format:

(17) $${\hat{b}}_{\text{ML-APES}}={\hat{b}}^{\left(1\right)}={\left\{F^{\left(1\right)}uu^{+}F^{(1)+}\right\}}_{\text{diag}}=(F^{\left(1\right)}u)⊙(F^{\left(1\right)}u)*,$$

where ⊙ defines the Shur-Hadamar (element vise) vector/matrix product. The algorithmic structure of the nonlinear (*i.e.*, solution-dependent) ML-APES estimator (17) guarantees positivity, but does not guarantee the consistency. Next, convergence enforcing regularization via performing projections onto convex solution sets (POCS) at each iteration step should be incorporated into the overall fixed-point iterative scheme for solving Equations (14,17) to guarantee the convergence in the considered here deficient-rank case. We defer the analysis of the consistency and convergence issues, as well as the POCS regularization with the relevant modifications of Equations (14,17) to the next Section proceeding now with the analysis of an alternative high-resolution SSP estimation approach based on the DEED regularization.

### 3.2. DEED Regularization Framework

The DEED regularization framework proposed and developed in [25,26,30,34] can be viewed as a problem-oriented formalization of the requirements to the signal/image processing/post-processing co-design aimed at satisfying the desirable properties of the reconstructed RS images, namely: (i) maximization of spatial resolution balanced with noise suppression, (ii) consistency, (iii) positivity, (iv) continuity and agreement with the data [2,25,34]. Within the general DEED framework [25,34], all these aspects are formalized via the design of balanced resolution-enhancement-over-noise-suppression SSP estimation techniques unified with the POCS regularization. Such unification-balancing is *descriptive* in the sense that the user/observer can induce the desirable metrics (geometrical) structures in the image/solution space, and next, specify the type, the order and the amount of the employed regularization via constructing the related resolution-enhancement-over-noise-suppression performance measures with adjustable balancing factors (regularization parameters). Different feasible assignments of such user-controllable “degrees of freedom” specify a family of the DEED-related techniques. For a detailed formalism of the DEED method we refer to [25,30] and for its implementation in a family of fixed-point iterative techniques to [26,34]. Here we provide a modification of the original DEED method [25,26] (in terms related to the presented above ML-APES strategy) to adapt it for the dynamic experiment design (DYED) regularization framework that we next develop in Section 5.

The DEED-optimal SSP estimate **b̂** is to be found as the POCS-regularized solution to the nonlinear equation [26]:

(18) $${\hat{b}}_{\text{DEED}}=𝒫{\left\{F_{\text{DEED}}uu^{+}F_{\text{DEED}}^{+}\right\}}_{\text{diag}}$$

where **F**_DEED_ represents the adaptive (*i.e.*, dependent on the SSP estimate **b̂**) matrix-form DEED solution operator and 𝒫 is the POCS regularization operator. Two fundamental issues constitute the benchmarks of the modified DEED estimator (18) that distinguish it from both the kernel algorithm (12) and the ML-APES method (17). First, the strategy for determining the DEED solution operator **F**_DEED_ in Equation (18) is reformulated in the minimum risk (MR)-inspired worst case statistical performance (WCSP) optimization setting [9,25] to provide robustness of the SSP vector estimates against possible model uncertainties, in particular, possible random distortions **Δ_S_** in the perturbed SFO matrix **S̃** = **S** + **Δ_S_** that result in multiplicative noise occurring in practical RS scenarios due to calibration errors and speckle noising effect [25,31,34]. The second issue relates to the problem-oriented co-design of the POCS regularization operator 𝒫 in Equation (18). Such co-design (that we perform in the next Section) is aimed at satisfying some intrinsic and desirable properties of the solution such as positivity, consistency, model agreement (e.g., despeckling with edge preservation), and convergence [2,37].

Following the DEED framework [25], the strategy for deriving the optimal SO **F**_DEED_ is formalized by the MR-WCSP optimization problem:

(19) $$F_{\text{DEED}}=\text{arg}\;\underset{F}{\text{min}}\;\underset{〈{\left\|\Delta_{\text{S}}\right\|}^{2}〉\leq \eta }{\text{max}}\{𝔕(F)\}$$

in which:

(20) $$𝔕(F)=\text{tr}\{〈(F\tilde{S}-I)A{\left(F\tilde{S}-I\right)}^{+}〉\}+\alpha \text{tr}\{〈FR_{n}F^{+}〉\}$$

represents the DEED objective function where the averaging in the first term (systematic risk component) is performed over the randomness of the distorted SFO **S̃** = **S** + **Δ_S_** with the uncertainty conditioned by the statistical bound $〈{\left\|\Delta_{S}\right\|}^{2}〉=〈\text{tr}\{\Delta_{S}\Delta_{S}^{+}\}〉\leq \eta$. The regularization parameter α and the invertible weight matrix **A** constitute the user controllable/adjustable “degrees of freedom”: α is viewed as a tolerance factor that balances the systematic risk component (specified by the first term in *ℜ* (**F**)) and the fluctuation risk component (specified by the second term in *ℜ* (**F**)) in the composite risk objective function (20), while **A** induces the weighted metrics structure in the systematic risk tr{〈(**FS̃** – **I**)**A**(**FS̃** – **I**)^+^〉} that measures “how far” is the DEED-optimal SO from the pseudo inverse to the uncertain SFO **S̃** in the averaged operator metrics induced by the employed weight matrix **A**. The solution to the conditioned optimization problem Equation (19) derived in the previous study [25,34] yields the DEED-optimal SO:

(21) $$F_{\text{DEED}}=F^{\left(2\right)}=KS^{+}R_{\sum }^{-1},$$

where $K={\left(S^{+}R_{\sum }^{-1}S+\alpha A^{-1}\right)}^{-1}$ defines the so-called reconstruction operator (with the regularization parameter α and stabilizer **A**^−1^), and $R_{\sum }^{-1}$ is the inverse of the diagonal loaded noise correlation matrix [34], **R**_Σ_ = **R**_Σ_ (β) = **R_n_** + β**I**. In the practical RS scenarios (and specifically, in all SAR imaging applications [1,3–7,25,32,34], *etc.*, it is a common practice to adopt the robust white additive noise model, *i.e.*, **R_n_** = *N*_0_**I**, attributing the unknown correlated noise component as well as the speckle to the composite uncertain noise term, **Δ_S_e** + **n**, in which case [25,34]:

(22) $$R_{\sum }=N_{\sum }I;\;\;\;\;\;\;\;N_{\sum }=N_{0}+\beta$$

with the composite noise power *N*_Σ_ = *N*_0_ + β, the additive observation noise power *N*_0_ augmented by the loading factor β = γη/α ≥ 0 adjusted to the regularization parameter α, the Loewner SFO ordering factor γ > 0 of the SFO **S** and the uncertainty bound η imposed by the conditional maximization in Equation (19) (see [25,34] for details). For these assumptions, the robust DEED-related SO becomes:

(23) $$F_{\text{DEED}}=F^{\left(3\right)}={\left(\Psi +\alpha N_{\sum }A^{-1}\right)}^{-1}S^{+}=KS^{+},$$

*i.e.*, a composition of the MSF operator **S**^+^ and the self adjoint reconstruction operator **K** = (**Ψ** + α*N*_Σ_**A^−^**^1^)^−1^ recognized to be the regularized inverse of the discrete-form ambiguity function (AF) matrix operator:

(24) $$\Psi =S^{+}S,$$

with the type and amount of regularization specified by the DEED degrees of freedom, **A** and α, respectively.

Putting **F**^(2)^, **F**^(3)^ in Equation (18) results in two POCS-regularized DEED-related SSP estimators that produce the SSP estimates defined as **b̂**^(2)^ and **b̂**^(3)^, respectively. Note that other feasible adjustments of the processing level degrees of freedom {α, *N*_Σ_, **A**} summarized in [26,34] for the robust RS adopted model (22) of the correlation matrix **R**_Σ_ specify the family of relevant POCS-regularized DEED-related (DEED-POCS) techniques (18) represented in the general form as follows:

(25) $${\hat{b}}^{\left(p\right)}=𝒫{\left\{D({\hat{b}}^{\left(p\right)})\right\}}_{\text{diag}}=𝒫{\left\{F^{\left(p\right)}uu^{+}F^{(p)+}\right\}}_{\text{diag}}=𝒫{\left\{K^{\left(p\right)}QK^{(p)+}\right\}}_{\text{diag}};\;\;\;p=1,\ldots ,P$$

where:

(26) $$Q=S^{+}uu^{+}S$$

defines the measurement statistics matrix independent on the solution **b̂**, and different reconstruction operators {**K**^(^*^p^*^)^; *p* = 1,…,*P*} specified for different feasible assignments to {α, *N*_Σ_, **A**} define the corresponding DEED-POCS estimators (25) with the relevant SOs’ {**F**^(^*^p^*^)^ = **K**^(^*^p^*^)^**S**^+^; *p* = 1,…,*P*}.

### 3.3. Relationship between DEED and ML-APES

The relationship between two high-resolution SSP estimators, the ML-APES (17) and the DEED-POCS (18), can now be established using the second equivalent form for representing the SO (16) given by (Appendix B, [21]):

(27) $$F_{\text{APES}}=F^{\left(1\right)}=\hat{D}S^{+}R_{u}^{-1}(\hat{b})=KS^{+}R_{n}^{-1},$$

which coincides with **F**_DEED_ = **F**^(2)^ specified by Equation (21) for the simplified scenario of completely certain SFO (**Δ_S_** = **0**), thus, **R**_Σ_ = **R_n_** = *N*_0_**I** in (22). Due to such structural algorithmic similarity, the DEED method (18) can be addressed as a regularized robust version of the APES approach (17) adapted to the uncertain RS scenarios.

## 4. Performance Guarantees

### 4.1. Consistency Guarantees

Following the DEED-POCS regularization formalism [26,34], the POCS-level regularization operator 𝒫 in Equation (18) could have a composite structure, *i.e.*, could be constructed as a composition of operators/projectors conditioned by non-trivial prior information that formalizes some desirable properties of the solution, e.g., positivity, consistency, *etc.* We specify such RS-adapted composite regularization in Section 4.4. In this Section, we are going to establish that to guarantee the consistency of the DEED-related estimator(s) (18), (25) the 𝒫 should incorporate a kernel-type WO 𝒲 (not necessarily isotropic) as a necessary requirement. To verify this consistency guarantee, we limit ourselves here with the relevant simplest regularization operator model, 𝒫 = 𝒲. Analysis of the consistency requires the hypothetical continuous asymptotes, $\underset{K\to \infty }{\text{lim}}{𝒫}_{𝔼(K)}=𝒤$, $\underset{M\to \infty }{\text{lim}}{𝒫}_{𝕌(M)}=𝒤$, the identity operators. Adopting these assumptions and white observation noise model, the DEED estimator given by Equation (25) can be expressed in the following generalized continuous functional form:

(28) $$\hat{b}(r)=𝒲|(𝒡u)(r){|}^{2}=\int_{R}W(r-r^{\prime }){\left|\int_{P}F(r^{\prime };p)u(p)dp\right|}^{2}dr^{\prime },$$

where *W*(**r**) is the functional kernel of the WO 𝒲, and *F*(**r;p**) represents the functional kernel of the continuous-form DEED-optimal SO 𝒡 = 𝒦𝒮^+^, a composition of the MSF operator 𝒮^+^ and the DEED-optimal reconstruction operator 𝒦 given by the continuous-form assymptotic to (23) (subscript _DEED_ is omitted to simplify the notations). To analyze the consistency of the estimator (28), one should consider the large measure [2] of the observation domain, 𝔐 = mes*P.* For the hypothetical asymptotes, 𝔐 → ∞, *N*_Σ_ → 0 the operator composition 𝒡𝒮 = 𝒦𝒮^+^𝒮 tends to the identity operator [2], in which case, the estimator (28) produces the degraded (smoothed by the WO) estimate of the SSP with the asymptotic bias $∥b-\underset{𝔐\to \infty }{\text{lim}}<{\hat{b}}_{𝔐}>{∥}^{2}=∥(𝒤-𝒲)b{∥}^{2}$ where $∥b{∥}^{2}={\left[b,b\right]}_{{𝕃}_{2}}=\int_{P}b(r)b*(r)dr$ defines the conventional (Lebesgue) squared norm in the 𝕃_2_ Hilbert space, and subscript 𝔐 indicates the measure of the observation domain for which the relevant estimate has been obtained. The ratio of the average fluctuation noise energy in the estimate (28) to the average fluctuation noise energy in the high-resolution sufficient statistics (SS), $v_{𝔐}=v_{𝔐}(r)=|\int_{P}F_{𝔐}(r;p)u(p)dp{|}^{2}$, is evaluated as follows:

(29) $$\xi (𝔐)=\frac{〈{\left\|𝒲v_{𝔐}-𝒲〈v_{𝔐}〉\right\|}^{2}〉}{〈{\left\|v_{𝔐}-〈v_{𝔐}〉\right\|}^{2}〉}=\frac{\text{tr}\{{𝒲𝒞}_{𝔐}{𝒲}^{+}\}}{\text{tr}\{{𝒞}_{𝔐}\}},$$

where 𝒞_𝔐_ defines the covariance operator of the SS, *v*_𝔐_(**r**), *i.e.*, the linear integral operator with the functional kernel cov{*v*_𝔐_(**r**)*v*_𝔐_(**r**′) = 〈*v*_𝔐_(**r**)*v*_𝔐_(**r**′)〉 – 〈*v*_𝔐_(**r**)〉 〈*v*_𝔐_(**r**′)〉. In the limiting case, 𝔐 → ∞, statistics *v*_𝔐_(**r**) becomes *δ* - correlated with the variance *b*^2^(**r**) [21]. Thus, one can evaluate the boundary value of the fluctuation noise ratio (29) as $\underset{𝔐\to \infty }{\text{lim}}\xi (𝔐)∼\underset{𝔐\to \infty }{\text{lim}}({𝔐}^{-1}\text{tr}\{{𝒲𝒲}^{+}\})$. Hence, the consistency requirement [36], $\underset{𝔐\to \infty }{\text{lim}}\xi (𝔐)=0$, is satisfied for any WO 𝒲 with the bounded operator norm, tr{𝒲𝒲^+^} < ∞ that provides $\underset{𝔐\to \infty }{\text{lim}}({𝔐}^{-1}\text{tr}\{{𝒲𝒲}^{+}\})=0$, that is for a *kernel* operator [2]. This restricts the class of admissible windows 𝒲 in Equation (28) by the kernel operators that in the engineering interpretation simply means that feasible windows must be restricted by a class of kernel-type filters.

*Remark* 1: The APES approach, as well as other ML-based high-resolution SSP estimators [21,24], *etc.*, do not imply regularizing windowing at all, *i.e.*, 𝒲 = *const*·𝒤 while the identity operator is not a kernel operator. Hence, in the uncertain scenarios with rank-deficient data covariance any ML-based approach inevitably produces an inconsistent estimate of the SSP. Technically it means that any ML-based resolution enhancement attempt should be combined with the relevant kernel windowing (not necessarily isotropic linear spatial smoothing) to guarantee the consistency.

### 4.2. Iterative Implementation

The next crucial performance issue relates to construction of convergent iterative scheme for efficient computational implementation of the POCS regularized DEED-related estimators. To convert such the technique to an iterative procedure we, first, transform the Equation (18) into the equivalent equation:

(30) $$𝒫\{\Phi_{D}\hat{b}\}=𝒫\{q\}$$

a numerical solution to which produces the desired SSP estimate **b̂**, where 𝒫{·} defines application of operator 𝒫 to the embraced vector quantity, **Φ_D_** is the solution-depended diagonal loaded point spread function (PSF) matrix operator:

(31) $$\Phi_{D}=\Phi_{D}(\hat{b})=(\Psi +N_{\sum }D^{-1}(\hat{b}))⊙(\Psi +N_{\sum }D^{-1}(\hat{b}))*,$$

constructed from the diagonal loaded AF matrix (24) via the Shur-Hadamar (element vise) product ⊙, and vector **q** represents the measurement statics vector

(32) $$q={\left\{Q\right\}}_{\text{diag}}={\left\{S^{+}YS\right\}}_{\text{diag}},$$

formed from the sampled data matrix **Y** (**Y** = **uu**^+^ in the considered above rank-1 data covariance matrix case) applying the MSF SO **S**^+^; *i.e.*, the **q** given by Equation (32) defines the low-resolution image formed by an RS radar or a fractional SAR imaging system that employs the conventional matched spatial processing algorithm. Fixing the Equation (30) at iteration [*i*], *i* = 1, 2, …, and inducing the “contractive mapping” term $({\hat{b}}_{\left[i+1\right]}-{\hat{b}}_{\left[i\right]})\underset{i\to \infty }{\to }0$, yields the progressive contractive mapping iterative scheme:

(33) $$\frac{1}{\tau }({\hat{b}}_{\left[i+1\right]}-{\hat{b}}_{\left[i\right]})+𝒫\{\Phi_{D[i]}{\hat{b}}_{\left[i\right]}\}=𝒫\{q\},$$

with the zero-step iteration **b̂**_[0]_ = **q** defined by Equation (32), in which the relaxation parameter τ and the regularization operator 𝒫 constitute the POCS regularization-level degrees of freedom that should be specified to guarantee the contractive mapping of Equation (33), hence, the convergence.

### 4.3. Convergence Guarantees

Following the POCS regularization formalism [2,26], the regularization operator 𝒫 could be constructed as a composition of projectors 𝒫*_n_* onto convex sets 𝔺*_n_*; *n* = 1,…, *N* with not empty intersection. Then for the composition of the relaxed projectors:

(34) $${𝒫}_{n}^{\lambda }=𝒤-\lambda_{n}({𝒫}_{n}-𝒤),$$

with the “speeding-up” regularization parameters {*λ_j_*}, the general-form POCS-regularized fixed-point iteration rule becomes:

(35) $${\hat{b}}_{\left[i+1\right]}={𝒫}_{N}^{\lambda }{𝒫}_{N-1}^{\lambda }\cdots {𝒫}_{1}^{\lambda }{\hat{b}}_{\left[i\right]}$$

and it is guaranteed to converge to a point in the intersection of the sets {𝔺*_n_*} provided 0 < *λ_n_* < 2 for all *n* in any order regardless of the initialization **b̂**_[0]_ that is a direct sequence of the fundamental theorem of POCS (Sec. 15.4.5, [20]) (see also (Sec. 6, [25]) and (Appendix B, [26]). In this study, for simplicity, we fix *λ_n_* = 1 for all *n* = 1,…, *N.* Also, any operator that acts in the same convex set (e.g., kernel-type WO) can be incorporated into such composite regularization operator 𝒫 [2]. Our next task is to make the use of the presented convergence enforcing POCS regularization paradigm employing some practical imaging radar/SAR-motivated considerations.

### 4.4. Resolution Preserving Anisotropic Windowing

The DEED-POCS framework offers a possibility to design the POCS regularization operator 𝒫 in such a way that to preserve high spatial resolution performances of the resulting DEED-related consistent SSP estimates. Following the VA-based image enhancement approach [16,20], this task could be performed via anisotropic image post-processing that in our statement implies anisotropic regularizing windowing over the properly constructed convex solution set in the image space. To proceed with the derivation of such a WO, in this paper, we incorporate the prior information on the desirable smoothness and geometrical properties of an image and its estimate by constructing the vector image/solution space 𝔹_(_*_K_*_)_ ∋ **b** as a *K*-D discrete-form approximation to the corresponding function image space 𝔹(*R*), in which the initial continuous SSP functions *b* (**r**), **r** ∈ *R* reside. To formalize the geometrical information on the image changes and simultaneously on the image edge changes over the scene frame, the metrics structure in 𝔹(*R*) must incorporate the image norm as well as the image gradient norm [2,3]. This is naturally to perform by adopting the so-called Sobolev metrics [21]:

(36) $${\left\|b\right\|}_{𝔼(R)}^{2}={\left[b,\;𝒨b\right]}_{{𝕃}_{2}(R)}=\int_{R}b(r)(\int_{R}M(r,\;r^{\prime })b(r^{\prime })dr^{\prime })*dr,$$

where *M* (**r**, **r**′) is the functional kernel of the metrics inducing operator 𝒨 constructed as a composition:

(37) $$𝒨=m^{\left(0\right)}𝒤+m^{\left(1\right)}\nabla_{r}^{2}=m^{\left(0\right)}𝒤+m^{\left(1\right)}\left\{D_{x}^{(1)+}D_{x}^{\left(1\right)}+D_{y}^{(1)+}D_{y}^{\left(1\right)}\right\}$$

in which $D_{x}^{\left(1\right)}=\partial /\partial x$ and $D_{y}^{\left(1\right)}=\partial /\partial y$ represent the first order differential operators with respect to the spatial variables *x* and *y*, respectively, $\nabla_{r}^{2}=D_{x}^{(1)+}D_{x}^{\left(1\right)}+D_{y}^{(1)+}D_{y}^{\left(1\right)}$ defines the Laplacian with respect to the 2-D space variable **r** = (*x*, *y*), and *m*^(0)^ and *m*^(1)^ are the nonnegative real-valued scalars that control the balance between two metrics measures in Equation (36). If *m*^(0)^ = 1, *m*^(1)^ = 0, then (36) reduces to the conventional Lebesgue 𝕃_2_ metrics in the Hilbert space 𝔹(*R*) that does not incorporate information on the image derivatives. In the opposite case *m*^(0)^ = 0, *m*^(1)^ = 1, the metrics Equation (36) transforms into the so-called Dirichlet variational functional [37] that controls only the gradient flow [2,37]. In the equibalanced case, *m*^(0)^ = *m*^(1)^ = 1, the same importance is assigned to the both metrics measures specified by the kernel metrics inducing operator 𝒨. Incorporation of such metrics inducing operator as the WO into the general DEED-optimal technique (28), *i.e.*, specifying 𝒲 = 𝒨, results in the required anisotropic kernel-type windowing because it controls not only the SSP (image) discrepancy measure but also its gradient flow over the scene in the Sobolev-type image/solution space 𝔹(*R*) ∋ *b*(**r**). In the next Section we show that due to the gradient-dependent anisotropy, such regularizing post-processing is aimed at edge preservation in the scene regions with high gradient contrast while performing smoothed windowing over the homogeneous image zones corrupted by speckle.

To proceed with designing the related WO adapted to the discrete problem model, the relevant 𝔹_(_*_K_*_)_ ∋ **b** as a *K*-D discrete-form approximation to 𝔹(*R*) ∋ *b*(**r**) has to be defined via specifying the corresponding metrics ${\left\|b\right\|}_{{𝔹}_{\left(K\right)}}^{2}=[b,\;Mb]$ with the metrics inducing matrix **M** constructed as a matrix-form approximation of 𝒨 given by Equation (37). For the adopted pixel-framed discrete image representation format {*b_k_* = *b*(*k_x_*, *k_y_*)}; *k* = (*k_x_*, *k_y_*); *k_x=_* 1,.., *K_x_*; *k_y=_* 1,.., *K_y_; k* = 1,…, *K* = *K_x_*×*K_y_*} this yields the desired metrics:

(38) $${\left\|b\right\|}_{{\text{B}}_{\left(K\right)}}^{2}=[b,\;Mb]=m^{\left(0\right)}\sum_{k_{x},k_{y}=1}^{K_{x},\;K_{y}}{\left(b(k_{x},\;k_{y})\right)}^{2}+m^{\left(1\right)}\sum_{k_{x},k_{y}=1}^{K_{x},K_{y}}{\left(b(k_{x},\;k_{y})-\frac{1}{4}\left(\begin{array}{l} b(k_{x}-1,\;k_{y})+b(k_{x}+1,\;k_{y})\\ +b(k_{x},k_{y}-1)+b(k,\;k_{y}+1) \end{array}\right)\right)}^{2}.$$

The second sum on the right hand side of Equation (38) is recognized to be a 4-nearest-neighbors difference-form approximation of the Laplacian operator $\nabla_{r}^{2}$ in Equation (37) [2,16]; hence, it represents the metrics measure of the high frequency spatial components in the discretized SSP that corresponds to is gradient variations. From Equation (38) we easily derive the corresponding metrics inducing matrix-form operator:

(39) $$M=m^{\left(0\right)}I+m^{\left(1\right)}\nabla^{2},$$

where ∇^2^ is the numerically approximated Laplacian operator $\nabla_{r}^{2}$. Application of such ∇^2^ to a vector **b** returns the vector ∇^2^**b** with elements defined by the terms in the second sum at the right hand side of Equation (38) ordered by multi index {*k* = (*k_x_*, *k_y_*) = 1,…, *K* = *K_x_* × *K_y_*} over the pixel-formatted 2-D frame. Also, in all applications below, we adopt the equibalanced metrics structure specifying *m*^(0)^ = *m*^(1)^ = 1.

Last, we restrict the solution subspace (the so-called active solution set or correctness set in the DEED terminology [25,34]) to the *K*-D *convex* set 𝔹_+_ ⊂ 𝔹_(_*_K_*_)_ of SSP vectors with nonnegative elements (as power is always nonnegative). This is formalized by specifying the projector 𝒫_+_ onto such convex set 𝔹_+_ *i.e.*, the POCS operator (as the positivity operator specifies POCS [2]) that has the effect of clipping off all the negative values. The composition:

(40) $$𝒫={𝒫}_{2}{𝒫}_{1},$$

defines the required composite POCS operator with the regularizing WO 𝒲 = 𝒫_1_ = **M** and 𝒫_2_ = 𝒫_+_ (in the function image space, 𝔹(*R*), the continuous-form generalization for the WO is given by 𝒲 = 𝒫_1_ = 𝒨).

## 5. DYED Regularization Framework

### 5.1. VA-Bases Dynamic Reconstructive Scheme

With the model (40), the discrete-form contractive progressive mapping iterative process (33) transforms into:

(41) $${\hat{b}}_{\left[i+1\right]}={\hat{b}}_{\left[i\right]}+\tau {𝒫}_{+}\{Mq-M\Phi_{D[i]}{\hat{b}}_{\left[i\right]}\};\;\;\;\;\;\;\;\;\;\;\;\;\;\;\;\;\;\;i=0,\;1,\;2,\;\ldots$$

initialized by the conventional low-resolution MSF image **b̂**_[0]_ = **q**. Some theoretical generalizations of Equation (41) for a hypothetical continuous STAP over the 2-D scene frame *R* ∋ **r** = (*x, y*) in “evolution time” are useful at this point for establishing the asymptotic dynamic adaptive reconstructive processing properties of the DEDR-VA approach.

Associating the iterations *i*, *i*+1,… with discrete “evolution time”, *i.e.*, *i* + 1 → *t* + Δ*t;i* → *t*;τ → Δ*t*, the Equation (41) can be rewritten in the “evolution” form:

(42) $$\left(1/\Delta t\right)\left[\hat{b}(t+\Delta t)-b(t)\right]={𝒫}_{+}\{Mq-M\Phi_{D}(t)\hat{b}(t)\}$$

with the corresponding dynamic scheme in hypothetical continuous evolution time (Δ*t* → *dt; t* +Δ*t* → *t* + *dt*) being:

(43) $$\frac{\partial \hat{b}(t)}{\partial t}={𝒫}_{+}\{Mq-M\Phi_{D}(t)\hat{b}(t)\},$$

where ∂**b̂**(*t*)/∂*t* represents the derivate with respect to the evolution time. Considering the continuous 2-D rectangular scene frame *R* ∋ **r** = (*x, y*) with the corresponding initial MSF scene image *q*(**r**) = *b̂*(**r**;0) and the “evolutionary” enhanced SSP estimate *b̂*(**r**; *t*), respectively, we proceed from (43) to the equivalent asymptotic dynamic scheme:

(44) $$\frac{\partial \hat{b}(r;t)}{\partial t}={𝒫}_{+}\{𝒨\{(q(r))\}-𝒨\{\int_{R}\Phi_{\hat{b}}(r,\;r^{\prime };t)\hat{b}(r^{\prime },\;t)dr^{\prime }\}\},$$

where Φ*_b̂_* (**r**, **r′**; *t*) represents the kernel PSF in evolution time *t* corresponding to the continuous-form dynamic generalization of the PSF matrix **Φ_D_**_[_*_i_*_]_ in Equation (31), and 𝒨 is the metrics inducing operator defined by Equation (37).

Three practically inspired versions of Equation (37) relate to three feasible assignments to the operator 𝒨. These are as follows:

1. 𝒨 = 𝒤 specifies the conventional Lebesgue metrics, in which case the evolution process (44) does not involve control of the image gradient flow over the scene.
2. $𝒨=\nabla_{r}^{2}$, *i.e.*, the Laplacian with respect to the space variable **r** = (*x*,*y*) specifies the Dirichlet variational metrics inducing operator, in which case, the right-hand side of (44) depends on the discrepancy between the corresponding Laplacian edge maps producing anisotropic gain. For short evaluation time intervals, such anisotropic gain term induces significant changes dominantly around the regions of sharp contrast resulting in edge enhancement [2,16].
3. $𝒨=m^{\left(0\right)}𝒤+m^{\left(1\right)}\nabla_{r}^{2}$ combines the Lebesgue and the Dirichlet metrics, in which case the Equation (44) is transformed into the VA dynamic process defined by the partial differential equation (PDE):
  (45) $$\frac{\partial \hat{b}(r;t)}{\partial t}={𝒫}_{+}\{c_{0}[q(r)-\int_{R}\Phi (r,\;r^{\prime };t)\hat{b}(r^{\prime };t)dr^{\prime }]+c_{1}\nabla_{r}^{2}\{q(r)\}-c_{2}\nabla_{r}^{2}\{\int_{R}\Phi (r,\;r^{\prime };t)\hat{b}(r^{\prime };t)dr^{\prime }\}\}.$$

For the purpose of generality, instead of two metrics balancing coefficients *m*^(0)^ and *m*^(1)^ we incorporated into the PDE (45) three regularizing factors *c*_0_, *c*_1_ and *c*_2_, respectively, viewed as VA-level user-controllable degrees of freedom to compete between smoothing and edge enhancement. Although due to the solution-depended nature the dynamic DEED-VA scheme in its continuous PDE form (45) cannot be addressed as a practically realizable procedure, the undertaken theoretical developments are useful for establishing the relationship between the general-form VA scheme (45) and the already existing dynamic image enhancement approaches [16–20].

### 5.2. VA-Relates Approaches

Different feasible assignments to the processing level degrees of freedom in the PDE (45) specify different VA-related procedures. Here beneath we consider the following ones:

- The simplest case relates to the specifications: *c*_0_ = 0, *c*_1_ = 0, *c*_2_ = const = – *c*, *c* > 0, and Φ(**r**, **r**′; *t*) = *δ*(**r** – **r**′) with excluded projector 𝒫_+_. In this case, the PDE (45) reduces to the *isotropic diffusion* (so-called *heat diffusion* [16]) equation $\partial \hat{b}(r;t)/\partial t=c\nabla_{r}^{2}\hat{b}(r;t)$ with constant (isotropic) conduction factor *c*. We reject the isotropic diffusion for the purposes of radar/SAR image processing because of its resolution deteriorating nature.
- The previous assignments but with the anisotropic factor, − *c*_2_ = *c*(**r**; *t*) ≥ 0 specified as a monotonically decreasing function of the magnitude of the image gradient distribution, *i.e.*, a function *c*(**r**,| ∇**_r_***b̂*(**r**; *t*)|) ≥ 0, transforms the Equation (45) into the celebrated Perona-Malik *anisotropic diffusion* method [16,18] $\partial \hat{b}(r;t)/\partial t=c(r;|\nabla_{r}\hat{b}(r;t)|)\nabla_{r}^{2}\hat{b}(r;t)$. Because the “model-free” assignment Φ(**r**,**r**′; *t*) = *δ*(**r** – **r**′) excludes the “model-based” (DEED regularization-based) SSP reconstruction, the anisotropic diffusion provides only partial despeckling of the homogeneous regions on the low-resolution MSF images preserving their edge maps.
- For the Lebesgue metrics specification *c*_0_ = 1 with *c*_1_ = *c*_2_ = 0, the PDE (45) involves only the first term at its right hand side. This case leads to the locally selective *robust adaptive spatial filtering* (RASF) approach investigated in details in our previous studies [25,34], where it was established that such the method provides satisfactory compromise between the resolution enhancement and noise suppression but suffers from low convergence rate.
- The alternative assignments *c*_0_ = 0 with *c*_1_ = *c*_2_ = 1 combine the isotropic diffusion with the anisotropic gain controlled by the Laplacian edge map. This approach addressed in [19,20] as a *selective information fusion* method manifests almost the same performances as the RASF method.
- The VA-based approach that we address here as the DEED-VA-fused DYED method involves all three terms at the right hand side of the PDE (45) with the equibalanced weights, *c*_0_ = *c*_1_ = *c*_2_ = *const* (one for simplicity), hence, it combines the isotropic diffusion (specified by the second term at the right hand side of Equation (45)) with the composite anisotropic gain dependent both on the evolution of the synthesized SSP frame and its Laplacian edge map. This produces a balanced compromise between the anisotropic reconstruction-fusion and locally selective image despeckling with edge preservation.

### 5.3. Numerical DEED-VA-Technique

The discrete-form approximation of the PDE (45) in “iterative time” {*i* = 0, 1, 2, …} yields the iterative numerical procedure:

(46) $${\hat{b}}_{\left[i+1\right]}={\hat{b}}_{\left[i\right]}+{𝒫}_{+}\{c_{0}\left(q-\Phi_{D[i]}{\hat{b}}_{\left[i\right]}\right)+c_{1}\nabla^{2}\{q\}-c_{2}\nabla^{2}\{\Phi_{D[i]}{\hat{b}}_{\left[i\right]}\}\};\;\;\;\;\;\;\;\;i=0,1,2\ldots ,$$

with the same MSF initialization **b̂**_[0]_ = **q**, where we have attributed the relaxation parameter τ to the corresponding VA regularization factors, for simplicity. The numerical Laplacian ∇^2^{·} applied to the embraces quantity is defined by the 4-nearest-neighbors difference-form approximation of the continuous Laplacian operator $\nabla_{r}^{2}$ specified by the terms in the second sum on the right hand side of Equation (38). Different feasible assignments to these degrees of freedom specify different related reconstruction techniques exemplified in the previous section, namely: isotropic diffusion, anisotropic diffusion, locally selective DEED-based reconstruction, and selective anisotropic reconstruction techniques. In particular, our DEED-VA-fused DYED method coincides with the previously developed conventional DEED regularization technique [26] in the case when no VA inspired discrepancy terms are adopted, *i.e.*, patting *c*_1_ = *c*_2_ = 0 in Equation (46). In contrary, the extended DEED-VA approach combines the VA-based isotropic diffusion with the anisotropic DEED reconstruction in a balanced fashion incorporating also the convergence enforcing POCS regularization. This not only speeds up the iterative process but provides perceptually enhanced imaging results as we illustrate in the comparative simulations presented in the next Section.

*Remark* 2: With the performed extension of the DEED regularization method into the unified DEED-VA framework, the warnings about the dynamic process (46) being ill conditioned do not apply, since the purpose of the two-level regularization (the DEED level and the VA level) is aimed at curing that same ill conditioning providing the POCS-regularized iterative DYED technique (46) converges to a point in the specified convex solution set 𝔹_+_. Nevertheless (as it is frequently observed with nonlinear iterative processes [2,36,37]) such nonlinear iterative procedure (46) may suffer from some numerical instabilities demonstrating only local convergence.

## 6. Numerical Simulations and Discussion

### 6.1. Simulation Experiment Specifications

In the simulation experiment, we considered a fractional SAR as a sensor system, analogous to a single look fraction of a multi look focused SAR [4,5,26,31]. The resolution properties of such the RS imaging system that employs the conventional MSF processing are explicitly characterized by the AF of the unit signal *S*(*t*,ρ;**r**) given by the composition [4,5,26]:

(47) $$\Psi (\Delta_{\tau },\;\Delta_{\theta })=C_{\Psi }\Psi_{r}(\Delta_{\tau })\Psi_{a}(\Delta_{\theta }).$$

**Figure 1.**
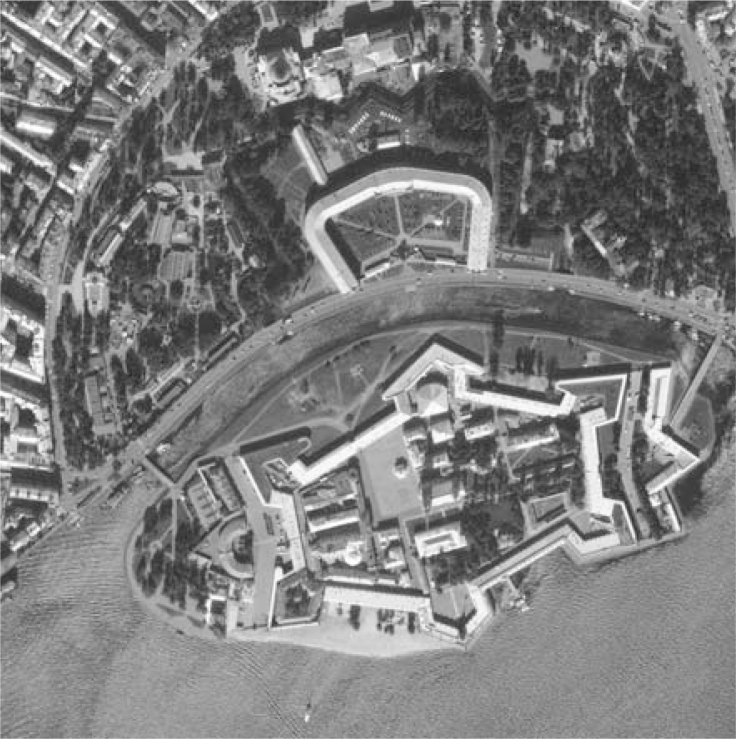
Original scene (not observable in the radar imaging experiment).

Here Ψ*_a_*(Δ*_θ_*) represents the azimuth AF over the azimuth angular spacing coordinate Δ_θ_ = arctan(Δ*x*/*r_s_*) related to the cross-range spacing Δ*x* = *x* − *x*′ between two scatterers at the particular slant range *r_s_* ; Ψ*_r_*(Δ*_τ_*) represents the range AF of the probe pulse signals as a function of the time delay variable Δ_τ_ = 2Δ*r_s_/c* related to the corresponding displacement Δ*r_s_* = *r_s_* – *r*′*_s_* of the scatterers along the slant range directions explicitly specified by the pulse modulation employed [4,26], *c* is the speed of light, and *C*_Ψ_ is a normalizing constant, not essential in the simulations [26]. To benefit from the range-angular AF factorization (47), the MSF images can be originally formed in the slant range planes (*r_s_*, θ) and then projected to the ground scene (*x*, *y*) = **r**∈*R* with the corresponding pixel spacing. For the Gaussian antenna tapering function [26], the related azimuth PSF Φ*_a_*(Δ*x*) expressed over the Cartesian coordinate *x* in the ground scene plane *R* is given by [26]:

(48) $$\Phi_{a}(\Delta x)={\left|\Psi_{a}(\Delta x)\right|}^{2}\approx {\left(e^{-\pi {\left(\Delta x\right)}^{2}/r_{s}^{2}{\left(\lambda_{0}/2L_{a}\right)}^{2}}\right)}^{2},$$

where *λ*_0_ is the wavelength of the radar signal transmitted, and *L_a_* is the effective (fractional) synthesized aperture max *L_a_* = *ψ_a_L*_max_, a fraction *ψ_a_* of the maximum focused synthesized antenna length *L*_max_ ≈ *λ*_0_*r_s_/L_A_* corresponding to the physical antenna with horizontal aperture *L_A_*. To be specific, the effective width *κ_a_* of the azimuth PSF Φ*_a_*(Δ*x*) is measured in pixels at the user selected threshold (e.g., *κ_a_*/2 at 0.5 from the maximum value Φ*_a_*(0) at the midrange 0 *r*_*s*_0__ [6,26]). In the same manner, *κ_r_* specifies the effective pixel width of the range PSF Φ*_r_*(Δ*y*) related to the discretized |sinc[2π*ψ_r_B*((2*r_s_* / *c*) – (2*r_s_′* / *c*))]|^2^-type *ψ_r_*-fractional range AF [6,22,26] in the slant range gates {*r_s_*, *r_s_*′} measured at the same user selected threshold (*κ_r_* /2 at 0.5 from the maximum value Φ*_r_*(0) [22,26]) where *B* represents the employed modulation signal bandwidth [4,6]. Because the DEED-VA-related fractional SAR image enhancement algorithms belong to the category of the MSF image post-processing techniques, the simulations were performed at the image processing level, *i.e.*, avoiding the SAR raw signal simulations [31]. We tested the (1024 × 1024)-pixel (*i.e.*, a large scale) scene image shown in Figure 1 in two hypothetical operational scenarios. The original scene was borrowed from the real-world high-resolution RS imagery [38]. Following [4,5,26,31] the degradations in the spatial resolution due to the fractional aperture synthesis mode were simulated via blurring the original image of Figure 1 with the range PSF Φ*_r_*(Δ*y*) along the *y*-axis and with the azimuth PSF Φ*_a_*(Δ*x*) along the *x*-axis, respectively. The degradations at the image formation level due to the propagation uncertainties were simulated using the statistical model of a SAR image defocusing [4,22,32]. The fractional resolution along the *x* and *y* scene coordinates were controlled by assigning different effective pixel widths *κ_r_* and *κ_a_* of the range and the azimuth PSFs and their varying over the scene that account to the range variation effect and uncompensated carrier trajectory deviations [4,22,26,31].

Next, to comply with the technically motivated MSF fractional image formation mode, the blurred scene image was degraded with the composite (signal-dependent) noise simulated as a realization of $\chi_{2}^{2}$ -distributed random variables with the pixel mean value assigned to the actual degraded scene image pixel. The simulation experiment compares three most prominent SAR-adapted enhanced imaging techniques, namely: the celebrated VA-based anisotropic diffusion method [16,18] specified in Section 5.2.(ii); the ML-APES method [24] detailed in Section 3.1, and the developed fused anisotropic DYED reconstruction method aggregated with the POCS regularization performed via Equation (46). The simulations were run for two hypothetical operational scenarios. The first one corresponds to the partially compensated defocusing errors [4,22]. In the second scenario, no autofocusing was assumed, thus the degradations encompass both uncontrolled SFO distortions and MSF mismatches attributed to “heavy” propagation medium perturbations [22,31], range migration effect [4] and uncompensated carrier trajectory deviations that may occur in much more severe operational scenarios [26,29,31]. For both scenarios, the simulations were run for different values of the composite signal-to-noise ratio (SNR) μ_SAR_ defined as the ratio of the average signal component in the degraded image **b̂**^(1)^ formed using the MSF algorithm (32) to the relevant composite noise component in that same speckle corrupted MSF image.

### 6.2. Performance Metrics

For objective evaluating of the reconstructive imaging quality, we have adopted two quality metrics traditionally used in image restoration/enhancement [2,3,11,37]. The first one evaluates the mean absolute error (*MAE*):

(49) $$MAE^{\left(p\right)}=10{\text{log}}_{10}\left\{\frac{1}{K_{x}K_{y}}\sum_{k_{x}=1}^{K_{x}}\sum_{k_{y}=1}^{K_{y}}\left|{\hat{b}}^{\left(p\right)}(k_{x},k_{y})-b(k_{x},k_{y})\right|\right\},$$

where {*b*(*k_x_*, *k_y_*)} represents the pixel values of the initial SSP and {*b̂*^(^*^p^*^)^ (*k_x_*, *k_y_*)} represents the pixel values of the SSP reconstructed applying the *p*th tested technique. In the performed simulations, *p* = 1 corresponds to the MSF algorithm (32), *p* = 2 corresponds to the VA-based anisotropic diffusion enhancement technique [16], *p* = 3 specifies the ML-APES method [24] resulting in the estimator (17), and *p* = 4 relates to the developed DYED-optimal fused DEED-VA algorithm (46). This metrics is well suitable for quantification of fine image reconstruction details, such as edge preservation (sharpening) and resolution of small targets on the extended scene [37].

The second employed metrics is the so-called improvement in the output signal-to-noise ratio (*IOSNR*) [2,3,29] measured via the ratio of the corresponding squared *l_2_* error norms:

(50) $${\text{IOSNR}}^{\left(p\right)}=10{\text{log}}_{10}\left({\left\|q-b\right\|}^{2}/{\left\|{\hat{b}}^{\left(p\right)}-b\right\|}^{2}\right),$$

where **b** represents the original SSP frame, **q** = **b̂**^(1)^ is the low-resolution speckle-corrupted image formed by a fractional SAR system that employs the conventional MSF method (32), and {**b̂**^(^*^p^*^)^} represents the SSP reconstructed from the corrupted MSF image **q** applying the *p*th enhanced imaging method from the simulated family, the same as in (49). The lower is the MAE and the higher is the IOSNR, the better is the image enhancement/reconstruction performed with the particular employed method.

### 6.3. Simulations Results and Discussions

**Figure 2.**
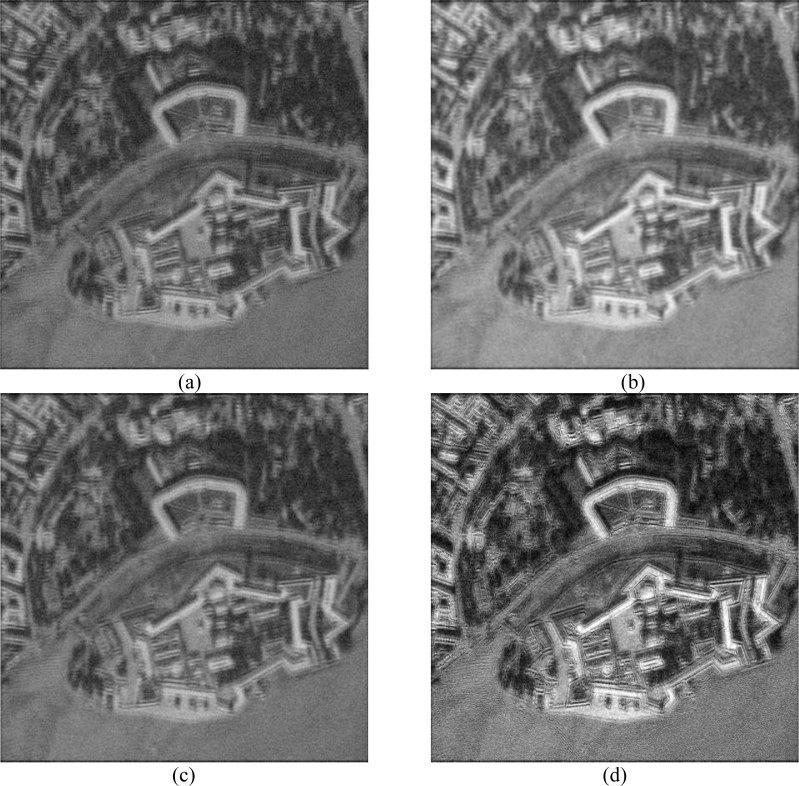
Simulation results for the first scenario: (**a**) degraded MSF image corrupted by composite noise (fractional SAR parameters: *κ_r_* = 10 pixels, *κ_a_* = 20 pixels, SNR μ_SAR_ = 15 dB); (**b**) the same scene image enhanced using the VA-AD technique; (**c**) result of reconstructive imaging performed with the ML-APES method (17); (**d**) the same image reconstructed applying the fused DYED technique (46), all after 30 performed iterations.

**Figure 3.**
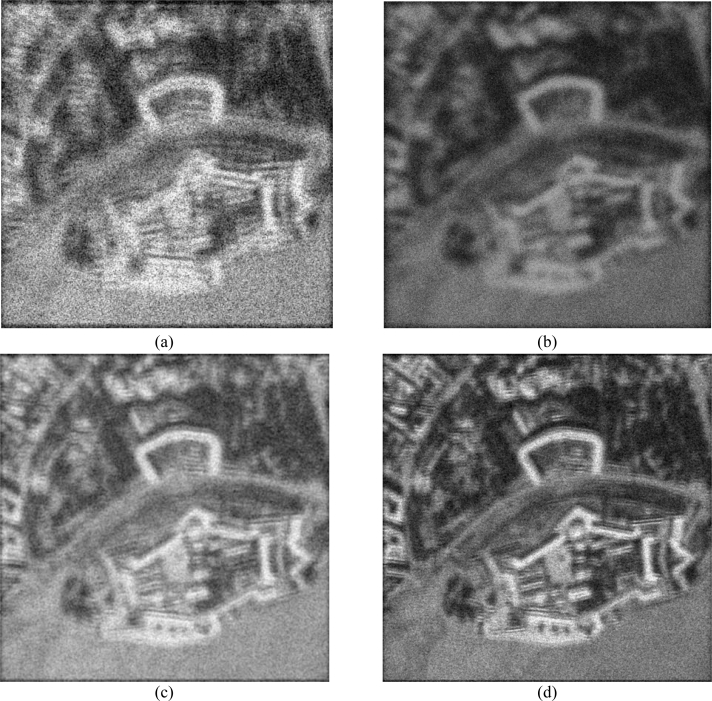
Simulation results for the second scenario: (**a**) degraded MSF image corrupted by composite noise (fractional SAR parameters: *κ_r_* = 20 pixels, *κ_a_* = 40 pixels, SNR μ_SAR_ = 10 dB); (**b**) the same image enhanced using the VA-AD technique; (**c**) result of reconstructive imaging performed with the ML-APES method (17); (**d**) the same image reconstructed applying the fused DYED technique (46), all after 30 performed iterations.

Figure 1 shows the original scene image (borrowed from the high-resolution RS imagery [38]) not observable with the simulated fractional SAR imaging systems. The images in Figures 2 and 3 present the results of image formation/enhancement applying different tested DEED-VA-related techniques in two operational scenarios as specified in the figure captions. Figures 2(a) and 3(a) demonstrate the images formed applying the conventional MSF algorithm. From these figures, one may easily observe that the MSF images suffer from imperfect spatial resolution due to the fractional aperture synthesis mode and composite observation/focusing mismatches and are corrupted by multiplicative signal-dependent noise. In the first scenario, the simulated degradations in the resolution are moderate over the range direction (*κ_r_* = 10) and significantly larger over the azimuth direction (*κ_a_* = 20). In the second scenario, the fractional SAR system suffers from much more severe degradations due to additional defocusing in both directions (*κ_r_* = 20; *κ_a_* = 40) and lower SNR.

**Figure 4.**
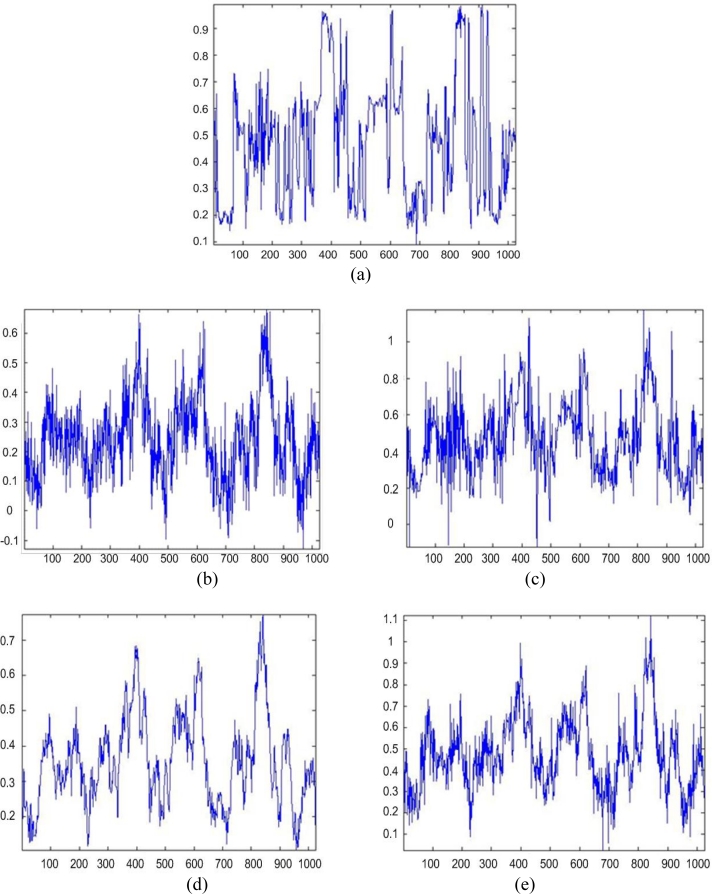
Comparative analysis of the 1-D imaging results: (**a**) the tested 1-D original image related to the 700th row vector from the 2-D scene of Figure 2; (**b**) degraded 1-D MSF image corrupted by composite noise (fractional SAR parameters: *κ_r_* = 10 pixels, *κ_a_* = 20 pixels, SNR μ_SAR_ = 15 dB); (**c**) the same 1-D image enhanced using the VA-AD technique; (**d**) 1-D image enhanced with the ML-APES method (17); (**e**) the same 1-D image reconstructed applying the fused DYED technique (46); iterative reconstructions (**c**), (**d**) and (**e**) are reported for the same 30 performed iterations.

**Figure 5.**
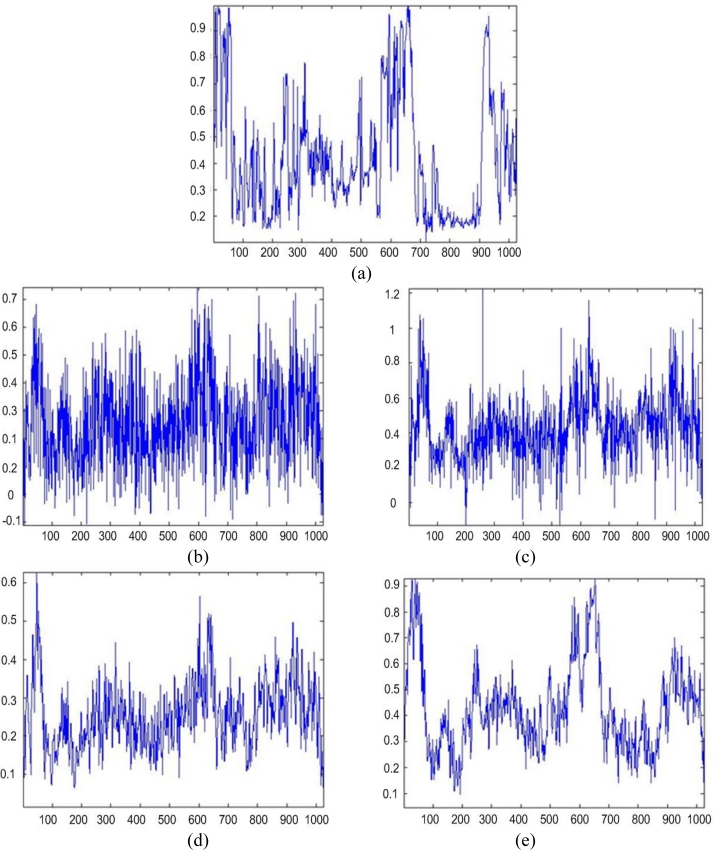
Comparative analysis of the 1-D imaging results: (**a**) the tested 1-D original image related to the 512th row vector from the 2-D scene of Figure 4; (**b**) degraded 1-D MSF image corrupted by composite noise (fractional SAR parameters: *κ_r_* = 20 pixels, *κ_a_* = 40 pixels, SNR μ_SAR_ = 10 dB); (**c**) the same 1-D image enhanced using the VA-AD technique; (**d**) 1-D image enhanced with the ML-APES method (17); (**e**) the same 1-D image reconstructed applying the fused DYED technique (46); iterative reconstructions (**c**), (**d**) and (**e**) are reported for the same 30 performed iterations.

**Figure 6.**
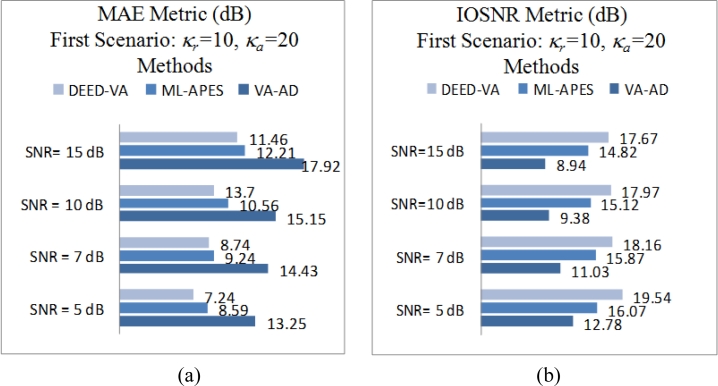
Quantitative reconstructive imaging performances for the first simulated operational scenario: (**a**) *MAE* metric; (**b**) *IOSNR* metric.

**Figure 7.**
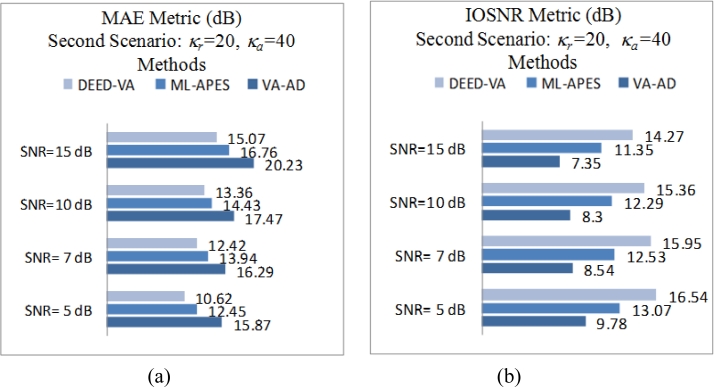
Quantitative reconstructive imaging performances for the second simulated operational scenario: (**a**) *MAE* metric; (**b**) *IOSNR* metric.

Next, Figures 2(b) and 3(b) show the images enhanced applying the anisotropic diffusion method [16,18]. The images reconstructed using the ML-APES method [24] are shown in Figures 2(c) and 3(c), and the corresponding images optimally reconstructed applying the DEED-VA-optimal technique (46) with the same equibalanced regularization factors *c*_0_ = *c*_1_ = *c*_2_ = 1, after the same 30 iterations are presented in Figures 2(d) and 3(d), respectively. Figures 4 and 5 present comparative results of reconstructive imaging in a 1-D format with the operational parameters and scenarios specified in the figure captions. Figures 6 and 7 report the quantitative performances evaluated via the two quality metrics (49) and (50) gained with three tested SSP estimation methods, namely: the VA-related anisotropic diffusion (VA-AD); the ML-APES and the DYED-optimal DEED-VA.

From the reported simulation results, the advantage of the well-designed imaging experiments (cases of the ML-APES and the optimal DEED-VA techniques) over the poorer design enhancement experiments (MSF and anisotropic diffusion (VA-AD) without ML-APES reconstruction) is evident for both scenarios. Due to the performed regularized inversions, the resolution was substantially improved. Quantitative performance improvement measures are reported in Figures 6 and 7 for the same 30 performed iterations.

**Figure 8.**
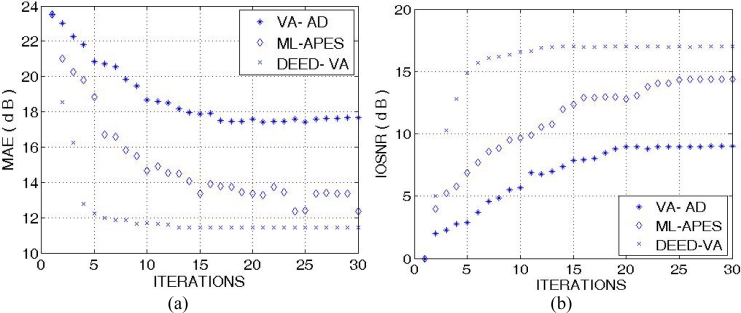
Convergence rates evaluated via: (**a**) *MAE* metric *versus* the number of iterations, and (**b**) *IOSNR* metric *versus* the number of iterations for three tested enhanced RS imaging methods, VA-AD, ML-APES and DEED-VA, respectively. The reported data correspond to the first tested operational scenario for the SNR μ_SAR_ = 15 dB.

The highest values of the *IOSNR*, as well as the lowest values of the *MAE*, were obtained with the DYED-optimal SSP estimator, *i.e.*, with the fused DEED-VA technique (46) adapted to the particular operational scenarios. Note that *IOSNR* (50) is basically a square-type error metric; thus, it does not qualify quantitatively the “delicate” visual features in the reconstructed RS images; hence, small differences in the corresponding *IOSNR*s reported in Figures 6 and 7. Furthermore, all the DYED-related estimators manifest the higher *IOSNR*s and lower *MAE*s in the case of higher SNR. Both the 2-D and 1-D RS imaging results are indicative of the superior qualitative reconstructive performances achieved with the high-resolution DYED-related estimators (17) and (46), while the DYED-optimal DEED-VA approach outperforms the ML-APES method. Last, in Figure 8, we report the convergence rates (specified via the dynamics of the corresponding *IOSNR* and *MAE* metrics *versus* the number of iterations) evaluated for the first test scenario for the same three dynamic enhancement/reconstruction techniques: VA-AD, ML-APES, and the developed DYED-optimal unified DEED-VA technique. The reported convergence rates are indicative of the considerably speeded-up performances manifested by the DEED-VA algorithm (46) that outperforms the most prominent existing ML-APES and anisotropic diffusion methods in the both quality metrics requiring 5–6 times less number of iterations to approach the same asymptotic convergence.

## 7. Conclusions

In this paper, we have addressed the unified DYED method for nonparametric high-resolution adaptive sensing of the spatially distributed scenes in the uncertain RS environment that extends the previously developed DEED regularization framework via its aggregation with the dynamic VA-based enhanced imaging approach. We have treated the RS imaging problem in an array radar/SAR adapted statement. The scene image is associated with the estimate of the SSP of the scattered wavefield observed through the randomly perturbed kernel SFO under severe snapshot limitations resulting in a degraded speckle corrupted RS image. The crucial issue in treating such a nonlinear ill-posed inverse problem relates to the development of a statistical SSP estimation/reconstruction method that balances the resolution enhancement with noise suppression and guaranties consistency, convergence and robustness of the resulting STAP procedures. In the addressed experiment design setting, all these desirable performance issues have been formalized via inducing the corresponding Sobolev-type metrics structure in the solution/image space, next, constructing the DEED-balanced resolution-enhancement-over-noise-suppression objective measures and, last, solving the relevant SSP reconstruction inverse problem incorporating the two-level regularization (the DEED level and the VA level, respectively). Furthermore, the incorporation of the second-level VA-based dynamic POCS regularization not only speeds up the related iterative processing procedures but provides also perceptually enhanced imagery. Also, the developed DYED method is user-oriented in the sense that it provides a flexibility in specifying some design (regularization) parameters viewed as processing-level degrees of freedom, which control the type, the order and the amount of the employed two-level regularization producing a variety of DYED-related techniques with different operational performances and complexity. Simulations verified that the POCS-regularized DEED-VA-optimal DYED technique outperforms the most prominent methods in the literature based on the ML and VA approaches that do not unify the DEED framework with the POCS-based convergence enforcing dynamic regularization in the corresponding applications.

## Acronyms

AF: Ambiguity function
ASF: Adaptive spatial filtering
DEED: Descriptive experiment design
DYED: Dynamic experiment design
EO: Equation of observation
ML: Maximum likelihood
MSF: Matched spatial filtering
PDE: Partial differential equation
POCS: Projections onto convex sets
PSF: Point spread function
RASF: Robust ASF
RS: Remote sensing
SAR: Synthetic aperture radar
SFO: Signal formation operator
SSP: Spatial spectrum pattern
STAP: Space-time adaptive processing
VA: Variational analysis
WO: Window operator
